# Long non-coding RNA LINC00649 regulates YES-associated protein 1 (YAP1)/Hippo pathway to accelerate gastric cancer (GC) progression via sequestering miR-16-5p

**DOI:** 10.1080/21655979.2021.1924554

**Published:** 2021-05-11

**Authors:** Hongyan Wang, Xin Di, Yingjie Bi, Shidong Sun, Tao Wang

**Affiliations:** a Department of Gastroenterology, PKUCare Luzhong Hospital, Zibo, Shandong, China; b Department of General Surgery, PKUCare Luzhong Hospital, Zibo, Shandong, China

**Keywords:** Gastric cancer, LINC00649, miR-16-5p, YES-associated protein 1, cancer biology

## Abstract

Although long non-coding RNA (LncRNA) LINC00649 is reported to be closely associated with acute myeloid leukemia (AML), prostate cancer and colorectal cancer, its role in regulating other types of cancer, such as gastric cancer (GC), has not been studied. This study analyzed the expression status of LINC00649 in GC tissues and cells by performing Real-Time qPCR analysis, and we found that LINC00649 tended to be enriched in cancerous tissues and cells but not in their normal counterparts, which were supported by the data from TCGA dataset. Next, by performing the gain- and loss-of-function experiments, we expectedly found that LINC00649 acted as an oncogene to accelerate GC cell proliferation, migration and epithelial-mesenchymal transition (EMT) *in vitro* and promote its tumorigenesis *in vivo*. Moreover, the online miRDB software predicted that miR-16-5p bound to both LINC00649 and 3ʹ untranslated region (3ʹUTR) of YAP1 mRNA, which were validated by the following dual-luciferase reporter gene system assay and RNA pull-down assay. Finally, we proved that LINC00649 exerted its tumor-promoting effects in GC by regulating the miR-16-5p/YES-associated protein 1 (YAP1)/Hippo pathway. Mechanistically, knock-down of LINC00649 suppressed YAP1 expressions by releasing miR-16-5p, resulting in the recovery of the Hippo pathway, which suppressed the expression levels of the downstream oncogenes, including EGFR, SOX2 and OCT4, leading to the inhibition of the malignant phenotypes in GC cells. In conclusion, this study, for the first time, evidenced that LINC00649 promoted GC progression by targeting the miR-16-5p/YAP1/Hippo signaling pathway, which provided potential diagnostic and therapeutic indicators for GC treatment for clinical utilization.

## GRAPHICAL ABSTRACT

**Figure:**
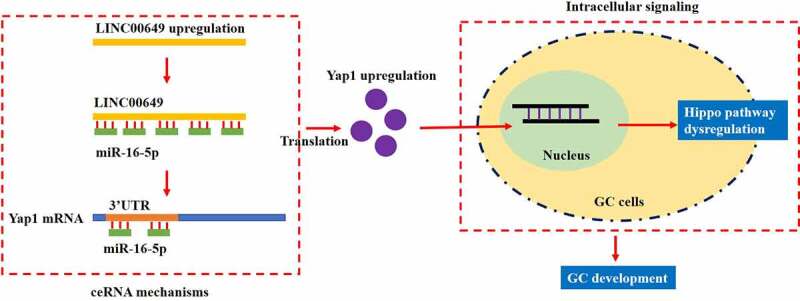

## Introduction

According to the published data, aberrant expression of long non-coding RNAs (LncRNAs) are closely relevant to cancer progression [1], including gastric cancer (GC) [2], and recent data indicated that targeting cancer associated LncRNAs might be effective to hamper the development of GC [3,4]. For example, Piao et al. reported that LncRNA PCGEM1 exerted its tumor-promoting effects in GC via aggravate cancer cells’ invasive and metastatic properties [4], and Pan et al. identified that silencing of LncRNA LIFR-AS1 hampered GC cell aggressiveness [3]. In addition, targeting LncRNAs also improved chemo-sensitivity in GC, and LncRNA UCA1 [5], LncRNA SNHG15 [6], and LncRNA HMGA1P4 [7] were considered as important regulators for cisplatin-resistance in GC cells. Thus, those existed data persuaded the researchers that identification of novel LncRNAs was feasible to develop effective treatment strategies for GC treatment in clinic [8,9]. Among all the cancer associated LncRNAs, LINC00649 gene located in chromosome 21 and contained 20 transcripts, which was poorly studied in the existed literatures, and our knowledge regarding to the biological functions of LINC00649 was seriously limited. To our knowledge, LINC00649 is identified as an important prognostic marker for acute myeloid leukemia (AML) [10,11], prostate cancer [12] and colorectal cancer [13], but its role in other types of cancers had not been reported.

The molecular mechanisms by which LncRNAs exerted their biological functions were complicated, which could be briefly divided into two branches, including epigenetic modification [14–16] and the classic competing endogenous RNA (ceRNA) mechanisms [17,18]. Based on the existed information from previous publications that LINC00649 mainly functioned as miRNA spongers [10,13], we conducted the associated bioinformatics analysis and screened out one of the downstream microRNAs (miRNAs), miR-16-5p, for further investigations. According to the previous publications, miR-16-5p functioned as tumor suppressor to restrain cancer development in multiple cancers, such as breast cancer [19], colorectal cancer [20], non-small cell lung cancer [21] and GC [22–24], and the associated LncRNAs, including LncRNA AGAP2-AS1 [25], LncRNA PVT1 [20], and LncRNA MEG3 [26] that could be targeted by miR-16-5p. However, up until now, no literatures reported the regulatory mechanisms of miR-16-5p and LINC00649, and the role of the LINC00649/miR-16-5p axis in regulating GC development had not been studied.

The classical Hippo pathway involved in regulating various diseases [27,28], including cancers [29,30]. To our knowledge, Hippo pathway sustained normal cellular functions to suppress cancer aggressiveness and development [29,30]. However, the normal cells acquired malignant phenotypes and turned into cancer cells when the Hippo pathway dysregulated [31,32], and recent publications reported that dysregulation of the Hippo pathway accelerated GC progression [33–35]. As previously described, the downstream Yes-associated protein 1 (YAP1) was proved as an oncogene to facilitate pathogenesis of multiple cancers [36], such as pancreatic cancer [37], lung cancer [38], and GC [39]. Specifically, Ajani et al. evidenced that YAP1 inhibition attenuated GC peritoneal metastasis [40], Wang et al, reported that YAP1 promoted malignancy and immunosuppression [41], and An et al. found that inactivation of YAP1 by MST4 kinase suppressed GC tumorigenesis [42]. Interestingly, the bioinformatics analysis predicted that there existed binding sites between miR-16-5p and 3ʹ untranslated regions (3ʹUTR), and some LncRNAs controlled YAP1 expression via regulating miRNAs in a ceRNA-dependent manner [43,44], which enlightened us that miR-16-5p might mediate the relationship between LINC00649 and YAP1.

Hence, by performing *in vitro* and *in vivo* experiments, this study managed to investigate the role of a novel LINC00649 in regulating GC pathogenesis, and we speculated that LINC00649 might regulate GC development via the miR-16-5p/YAP1/Hippo signaling pathway. Our study will identify novel biomarkers for GC diagnosis and treatment, which was of clinical significance.

## Materials and methods

### Clinical specimens

The 54 paired cancerous and non-cancerous tissues were collected from GC patients by surgical resection in PKUCare Luzhong Hospital from 2018 to 2020, which were immediately stored at −70°C conditions for further analysis. The GC patients did not accept any other therapies, such as chemotherapy and radiotherapy, before surgery. All the clinical experiments were approved by the Ethics Committee of PKUCare Luzhong Hospital, and all the participants had signed the inform consent forms.

### Cell culture and vectors transfection

The GC cells (MGC-803 and SGC-7901) and a normal gastric epithelial cell line GES-1 were bought from American Type Culture Collection (ATCC, USA), which were selected in this study in keeping with the previous publications. The above cells were cultured in our lab in the Dulbecco’s modified Eagle’s medium (DMEM, Hyclone, USA) with 10% fetal bovine serum (FBS, Gibco, USA), in an incubator with the standard culture condition with 5% CO_2_ at 37°C. The overexpression and downregulation vectors for LINC00649, miR-16-5p mimic and inhibitor, and YAP1 overexpression vectors were designed as previous described [10,19,45], which were synthesized and transfected into the GC cells by a commercial third-party company (Sangon Biotech, Shanghai, China) via using a Lipofectamine 2000 reagent (Invitrogen, USA) in keeping with the manufacturer’s protocol. The transfection efficiency was determined by using the Real-Time qPCR analysis.

### Real-Time qPCR

The clinical tissues and cells were prepared, and the total RNA was extracted by using the commercial TRIzol reagent (Takara, Japan), and the expression levels of the cancer-associated genes were measured by using the following Real-Time qPCR analysis according to previous work [13]. Briefly, the total RNA was examined by 2% agarose gel electrophoresis, and the expression levels of the target genes were examined by using the One-Step TB Green ^TM^ PrimeScript ^TM^ RT-PCR kit purchased from Takara (Japan) in keeping with the manufacturer’s protocol. According to the information provided by the previous publications [13,20,39,43,44], the primer sequences for LINC00649, miR-16-5p, YAP1 mRNA, U6, GAPDH, EGFR mRNA, SOX2 mRNA and OCT4 mRNA were designed.

### Western Blot analysis

The total proteins were extracted from the clinical tissues and commercial cell lines by using the RIPA lysis buffer (Beyotime, Shanghai, China), which were subsequently separated by the 10% SDS-PAGE and the target protein bands were transferred onto the PVDF membranes (Millipore, USA) according to the proteins’ molecular weight. The PVDF membranes were blocked by 4% skim milk, and the membranes were incubated with the primary antibodies against YAP1 (1:1500, Abcam, UK), GAPDH (1:2000, Abcam, UK), N-cadherin (1:1500, Abcam, UK) and E-cadherin (1:2000, Abcam, UK) at 4°C overnight. Then, the membranes were added with the secondary antibodies (1:5000, Cell Signaling Technology, USA) for 2 h at room temperature, and the protein bands were visualized by ECL system (Elabscience, Wuhan, China).

### MTT assay

The GC cells were pre-transfected with different vectors, and the cells were cultured in the 96-well plates in the incubator for 0 h, 24 h, 48 h and 72 h, respectively. Next, the MTT reaction solution was incubated with the cells at the concentration of 20 μl per well for 4 h to generate the formazan, which were subsequently diluted by using the DMSO. The plates were vortexed and a microplate reader (ThermoFihser Scientific, USA) was used to measure the optical density (OD) values in each well, which could be used to evaluate relative cell proliferation abilities.

### Trypan blue staining assay

The GC cells with differential vectors transfection were cultured under standard culture conditions for 0 h, 24 h, 48 h and 72 h, respectively, and the trypan blue staining assay was performed to evaluate cell viability. Specifically, the cells were stained with trypan blue staining buffer for 15 min at 37°C, and a light microscope was used to observe and count the dead blue cell numbers. Cell viability was calculated according to the ratio of dead blue cells and total cells.

### Flow cytometry (FCM)

The GC cells were stained with Annexin V-FITC and PI for 40 min at room temperature without light exposure, and a flow cytometer (BD Bioscience, USA) was used to measure the apoptosis ratio of the GC cells, and the both the Annexin V-FITC- and PI-positive cells were regarded as apoptotic cells, which were subsequently analyzed by using the FlowJo VX software.

### Dual-luciferase reporter gene system assay

The targeting sites in LINC00649, miR-16-5p and YAP1 mRNA were predicted by using the online miRDB (http://mirdb.org/) and starBase software (http://starbase.sysu.edu.cn/), which were validated by the following dual-luciferase reporter gene system assay. The binding sites in LINC00649 and YAP1 mRNA were mutated and were cloned into the luciferase reporter by a commercial third-party company (Sangon Biotech, China), which were co-transfected with miR-16-5p mimic into the GC cells by using the Lipofectamine 2000 reagent (Invitrogen, USA) for 48 h, and the Dual-Luciferase Reporter Assay Kit (Promega, USA) was used to measure relative luciferase activities.

### RNA pull-down assay

The predicted targeting sequences in LINC00649 and YAP1 mRNA were used to construct biotinylated-LINC00649 and biotinylated-YAP1 probes, and the oligo probe (5ʹ-TAT CAC GTA GCC GTT GCA TTT GCC GTA GCC CTG TGG GCC-3ʹ) was used as control. The GC cells were lysed, and the lysates were incubated with the above probes to form the LINC00649-miR-16-5p and YAP1-miR-16-5p complexes, which were subsequently pulled down by using the streptavidin-coated magnetic beads. Then, the miR-16-5p enrichment was quantified by using the following Real-Time qPCR analysis.

### Animal experiments

The MGC-803 cells were used for *in vivo* animal experiments. Briefly, the female BALB/c nude mice with 4-week-old were purchased, and the mice were maintained under the specific-pathogen-free (SPF) conditions with 12 h light-dark cycle. The MGC-803 cells with or without LINC00649-deficiency were subcutaneously injected into the right dorsal flank of the mice legs, at day 30 post-injection, the mice were anesthetized by using the sodium barbital at the concentration of 2.5 mg/kg. The mice were sacrificed by using the head-removal method once the heart beats of the mice could not be detected, and the mice tumor were removed and weighed to evaluate tumorigenesis of the MGC-803 cells *in vivo*. All the animal experiments were approved by the Ethics Committee of PKUCare Luzhong Hospital.

## Data analysis

All the data were collected and presented as Means ± Standard Deviation (SD). By performing SPSS18.0 software, the means from two groups were compared by the Student’s t-test, and one-way ANOVA analysis was performed to compare the means from multiple groups. The correlations of LINC00649, miR-16-5p and YAP1 mRNA were analyzed by Pearson Correlation Analysis. *P* < 0.05 was regarded as statistical significance, which was marked by ‘*’.

## Results

### The expression status of LncRNA LINC00649, miR-16-5p and YAP1 in GC tissues and cells

**Figure 1.**
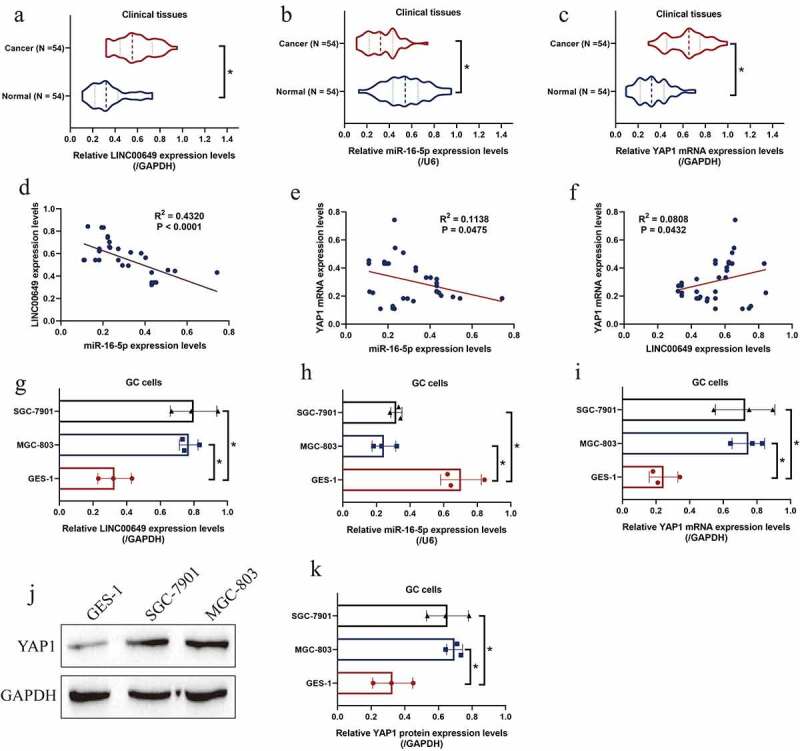
LINC00649, miR-16-5p and YAP1 were relevant to GC malignancy. The expression status of (a) LINC00649, (b) miR-16-5p, and (c) YAP1 mRNA in the clinical specimens were quantified by using the Real-Time qPCR analysis. (d-f) The correlations of LINC00649, miR-16-5p and YAP1 mRNA in the GC tissues were analyzed by pearson correlation analysis. (g-i) Real-Time qPCR was used to detect LINC00649, miR-16-5p and YAP1 mRNA expression levels in GC cells, and (j, k) the YAP1 protein levels in the cells were measured by using the Western Blot analysis. Each experiment had three individual repetitions, and *P < 0.05 was regarded as statistical significance

Based on the previous literatures [46,47], we initially examined the expression status of LINC00649, miR-16-5p and YAP1 at both clinical and cellular levels. The 54 paired cancerous and the adjacent non-cancerous tissues were collected from GC patients for further analysis, and the Real-Time qPCR was performed to examine the expression levels of LncRNA LINC00649 (Figure 1(a)), miR-16-5p (Figure 1(b)) and YAP1 mRNA (Figure 1(c)) in the above clinical tissues. As shown in Figure 1(a-c), LncRNA LINC00649 and YAP1 mRNA were aberrantly high-expressed, while miR-16-5p tended to be downregulated in the GC tissues, in contrast with the normal tissues. Also, the Pan-cancer analysis results supported that the expression levels of LncRNA LINC00649 (Figure S1A) and YAP1 mRNA (Figure S1B) were higher in the cancerous tissues (N = 375) than that of the normal tissues (N = 30) collected from patients with stomach adenocarcinoma (STAD), and STAD patients with high-expressed YAP1 tended to have a worse prognosis (Figure S1C). Next, we analyzed their correlations by performing the Pearson Correlation Analysis, and the results expectedly showed that miR-16-5p negatively correlated with both LncRNA LINC00649 (Figure 1(d)) and YAP1 mRNA (Figure 1(e)), but LncRNA LINC00649 and YAP1 mRNA showed positive correlations (Figure 1(f)) in the GC tissues. In addition, we evidenced that LncRNA LINC00649 (Figure 1(g)) and YAP1 (Figure 1(i-k)) were upregulated, while miR-16-5p was downregulated (Figure 1(h)) in the GC cells (MGC-803 and SGC-7901) but not in the normal GES-1 cells, which were in consistent with our clinical results.

### *LncRNA LINC00649 accelerated GC progression* in vitro *and* in vivo

**Figure 2.**
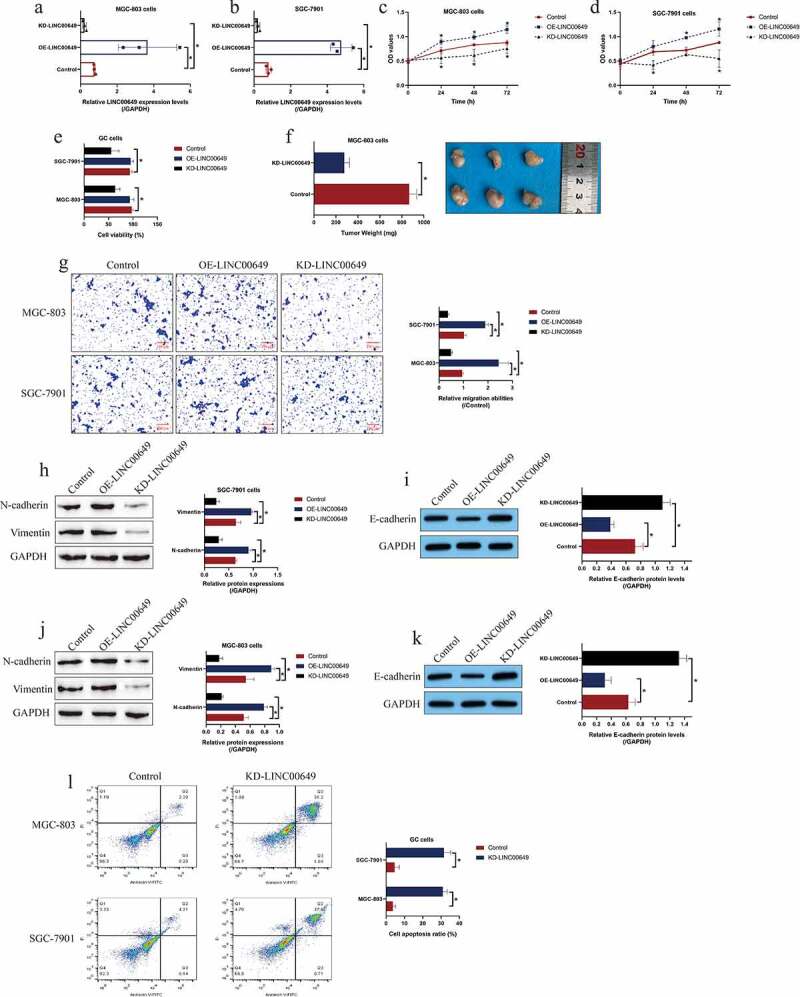
Targeting the LINC00649 hindered cell proliferation, viability, EMT and tumorigenesis in GC. (a, b) The transfection efficiency for LINC00649 overexpression and downregulation vectors were determined by Real-Time qPCR analysis. (c, d) MTT assay was performed to examine cell proliferation abilities. (e) Cell viability was determined by trypan blue staining assay. (f) *In vivo* tumors were obtained and weighed to evaluate tumorigenesis of MGC-803 cells. (g) Transwell assay was performed to determine cell migration abilities. (h-k) The EMT-associated biomarkers in GC cells were examined by using the Western Blot analysis. (l) The GC cells were stained with Annexin V-FITC and PI to examine cell apoptosis ratio. Each experiment had three individual repetitions, and *P < 0.05 was regarded as statistical significance

The biological functions of LINC00649 in regulating GC development had not been investigated in the existed studies, thus, we explored this issue by conducting further gain- and loss-of-function experiments. To achieve this, LINC00649 was respectively overexpressed and silenced in the GC cells by using their corresponding gene manipulating vectors, and the transfection efficiency was shown in Figure 2(a-b). Then, the MTT assay was performed, and the results suggested that LINC00649 overexpression promoted GC cell proliferation a time-dependent manner, which were suppressed by LINC00649 silence (Figure 2(c, d)). Similarly, knock-down of LINC00649 decreased cell viability from 98% to 56% (Figure 2(e)), which were in consistent with the MTT assay. Also, the xenograft tumor-bearing mice models were established by using the MGC-803 cells, and those data supported that LINC00649 ablation restrained tumor growth *in vivo* (Figure 2(f)). In addition, the Transwell assay results in Figure 2(g) indicated that LINC00649 positively regulated GC cell migration, and further Western Blot analysis results supported that overexpressed LINC00649 upregulated N-cadherin and Vimentin, and downregulated E-cadherin to trigger epithelial-mesenchymal transition (EMT) process in GC cells, while silencing of LINC00649 had opposite effects (Figure 2(h-k)). Moreover, by performingthe FCM assay, we provided evidence that knock-down of LINC00649 promoted cell apoptosis in GC cells (Figure 2(l)).

### The regulatory mechanisms of LncRNA LINC00649, miR-16-5p and YAP1 in GC cells

**Figure 3.**
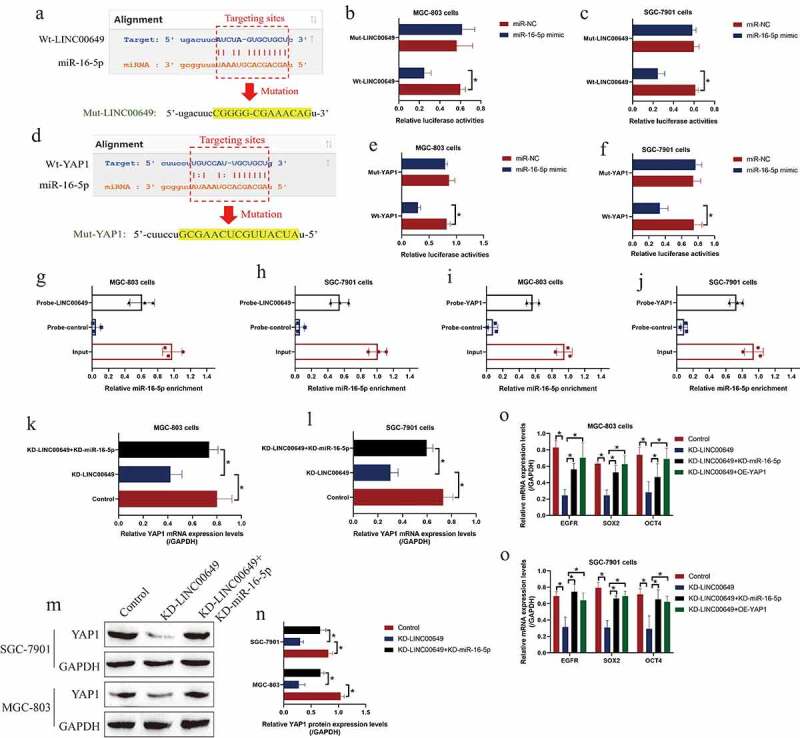
MiR-16-5p was able to target both LINC00649 and YAP1 in GC cells. (a, d) The tarting sites among LINC00649, miR-16-5p and YAP1 were predicted by miRDB software, and (B-C, E-F) the above targeting sites were validated by performing the following dual-luciferase reporter gene system assay. (g-j) RNA pull-down assay was performed to evaluate the binding abilities of miR-16-5p with LINC00649 and YAP1. (k, l) Real-Time qPCR and (m, n) Western Blot validated that LINC00649 positively regulated YAP1 via sponging miR-16-5p. (o, p) The mRNA levels of EGFR, SOX2 and OCT4 were examined by using the Real-Time qPCR analysis. Each experiment had three individual repetitions, and *P < 0.05 was regarded as statistical significance

Next, the regulatory mechanisms among LINC00649, miR-16-5p and YAP1 in GC cells were investigated. The bioinformatics analysis predicted that there existed targeting sites in miR-16-5p with both LINC00649 and 3ʹ UTR of YAP1 mRNA, according to the principle of competing endogenous RNA (ceRNA) networks, we conjectured that miR-16-5p might serve as a ‘bridge’ to combine LINC00649 and YAP1. To validate the above hypothesis, the targeting sites in LINC00649 and YAP1 mRNA were mutated and named as Mut-LINC00649 and Mut-YAP1, and the corresponding wild-type sequences were described as Wt-LINC00649 and Wt-YAP1, the above sequences were subsequently cloned into the luciferase vectors (Figure 3(a, d)). As expected, our data suggested that miR-16-5p mimic specifically decreased luciferase activities in the GC cells co-transfected with Wt-LINC00649 (Figure 3(b, c)) and Wt-YAP1 (Figure 3(e, f)), instead of their wild-type counterparts. Besides, the targeting sequences in LINC00649 and YAP1 mRNA were used to generate biotin-labeled LINC00649 and YAP1 probes, and the following RNA pull-down assay supported that miR-16-5p could be significantly enriched by the above probes in GC cells (Figure 3(g-j)). Moreover, knock-down of LINC00649 suppressed YAP1 expression at both transcriptional (Figure 3(k, l)) and translated (Figure 3(m, n)) levels, which were rescued by silencing miR-16-5p, indicating that LINC00649 regulated YAP1 expressions in a miR-16-5p-dependent manner. Furthermore, we noticed that LINC00649 ablation modulated the miR-16-5p/YAP1 axis to decrease the mRNA levels of EGFR, SOX2 and OCT4 (Figure 3(o, p)), which were the downstream targets of YAP1/Hippo pathway.

### LncRNA LINC00649 knockdown suppressed GC development via the miR-16-5p/YAP1 pathway

**Figure 4.**
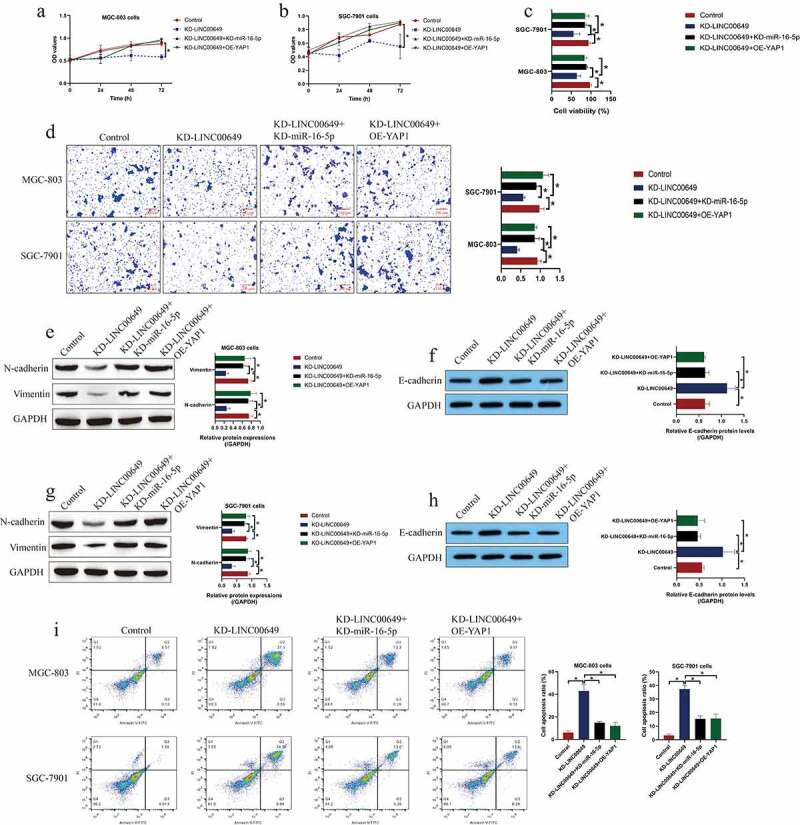
Silencing of LINC00649 reversed the malignant phenotypes in GC cells by regulating the miR-16-5p/YAP1 axis. (a, b) MTT assay was performed to evaluate cell proliferation abilities. (c) Trypan blue staining assay was performed to detect cell viability. (d) Cell migration abilities were evaluated by using the Transwell assay. (e-h) The expression levels of N-cadherin, Vimentin and E-cadherin were examined by Western Blot analysis. (i) FCM assay was used to evaluate cell apoptosis ratio in the GC cells. Each experiment had three individual repetitions, and *P < 0.05 was regarded as statistical significance

Since we had identified a novel LINC00649/miR-16-5p/YAP1 signaling cascade in GC cells, the following experiments were conducted to investigate the role of this axis in regulating GC development. The LINC00649 knockdown vectors, miR-16-5p inhibitor and YAP1 overexpression vectors were delivered into the GC cells, which were subsequently divided into four groups, including Control, KD-LINC00649 group, KD-LINC00649 + KD-miR-16-5p group, and KD-LINC00649 + OE-YAP1 group. As expectedly, the inhibiting effects of LINC00649 downregulation on cell proliferation (Figure 4(a, b)) and viability (Figure 4(c)) were abrogated by both silencing miR-16-5p and upregulating YAP1. Also, the Transwell assay was conducted, and we evidenced that knock-down of LINC00649 suppressed cell migration by modulating the miR-16-5p/Yap1 axis (Figure 4(d)), and the following Western Blot analysis evidenced that that the regulating effects of LINC00649 ablation on N-cadherin, E-cadherin and Vimentin were reversed by both miR-16-5p silence and YAP1 upregulation (Figure 4(e, h)). Finally, we performed the FCM assay, and the results in Figure 4(i) hinted that downregulated miR-16-5p and overexpressed YAP1 restored LINC00649 ablation-induced cell apoptosis in GC cells.

## Discussion

Although emerging data suggested that targeting LncRNAs was effective to hamper cancer progression and improve drug-resistance in GC, and multiple LncRNAs had been identified as diagnostic, therapeutic and prognostic biomarkers for GC, the involvement of a novel LncRNA LINC00649 in regulating GC pathogenesis had not been studied. Based on the information provided by the previous literatures, LINC00649 acted as an oncogene to facilitate the development of acute myeloid leukemia (AML) [10,11], prostate cancer [12] and colorectal cancer [13], but it was still unclear whether LINC00649 modulated GC progression in a similar manner. Hence, by conducting clinical and preliminary experiments, this study assured that LINC00649 also exerted its tumor-promoting effects in GC, which were in consistent with the previous publications in other types of cancers [10–13]. Specifically, our data evidenced that LINC00649 tended to be enriched in GC tissues and cells, in contrast with the normal counterparts, and LINC00649 positively regulated GC cell proliferation, migration, epithelial-mesenchymal transition (EMT) and tumorigenesis *in vitro* and *in vivo*, hinting that LINC00649 served as an oncogene for GC.

LncRNAs exerted their biological functions through multiple branches of mechanisms, including epigenetic modification [14–16] and the classic competing endogenous RNA (ceRNA) mechanisms [17,18], and the existed data showed that LINC00649 mainly functioned as miRNAs sponger to achieve their biological functions [10,13]. Thus, by conducting bioinformatic analysis and the following experiments, we firstly validated that LINC00649 sponged miR-16-5p in GC cells in a ceRNA-dependent manner, which had not been reported elsewhere. In addition, the expression levels of LINC00649 and miR-16-5p exhibited negative correlations in GC tissues, and miR-16-5p was downregulated in GC tissues and cells, which were in consistent with the previous data [22–24]. As previously described, miR-16-5p played a tumor-suppressing effects to slow down cancer progression [19–21], including GC [22–24], which evidenced the opposite effects of LINC00649 and miR-16-5p in regulating GC development. Thus, we performed further gain- and loss-of-function experiments, and expectedly found that knock-down of LINC00649 suppressed GC pathogenesis via releasing miR-16-5p, which were partially supported by the previous publications [10–13,22–24].

The YAP1/Hippo signaling pathway was closely associated with GC progression, and YAP1-induced dysregulation of Hippo pathway contributed to cancer development [36]. According to the previous data, YAP1 served as an oncogene in GC, and targeting YAP1 was effective to restrain cancer progression [37–39], which were supported by our data that YAP1 was high-expressed in GC tissues and cells, and GC patients with high-expressed were prone to have a worse prognosis. In addition, recent data hinted that YAP1 could be epigenetically silenced by its upstream miRNAs [48,49], and we surprisingly evidenced that miR-16-5p targeted the 3ʹ UTR of YAP1 mRNA for its inhibition and degradation. Moreover, knock-down of LINC00649 downregulated YAP1 at both transcriptional and translational levels, and decreased the expression levels of EGFR, SOX2 and OCT4 to recover the normal functions of the Hippo pathway via releasing miR-16-5p in GC cells, suggesting that LINC00649 regulated the YAP1/Hippo signaling pathway by targeting miR-16-5p. Then, we evidenced that the inhibiting effects of LINC00649 ablation on the malignant phenotypes in GC cells were abrogated by upregulating YAP1.

## Conclusions

We identified a novel LINC00649/miR-16-5p/YAP1 axis that regulated Hippo pathway to facilitate the development of GC. Specifically, LINC00649 sponged miR-16-5p to upregulate YAP1, resulting in the dysregulation of the downstream Hippo pathway, which further led to GC progression. Our study provided new cancer associated biomarkers for GC diagnosis and treatment, however, future work is still needed to investigate the role of above genes in regulating drug resistance in GC.

## Supplementary Material

Supplemental Material

## Highlights

1. LINC00649 promotes cell proliferation, migration and EMT in GC.
2. LINC00649 positively regulates YAP1 expressions in GC cells by sponging miR-16-5p.
3. Silencing of LINC00649 suppresses GC malignancy via modulating the miR-16-5p/YAP1 axis.
