# Supplementary material for: Long non-coding RNA LINC00649 regulates YES-associated protein 1 (YAP1)/Hippo pathway to accelerate gastric cancer (GC) progression via sequestering miR-16-5p
Source: Bioengineered. 2021 May 11;12(1):1791–802. doi: 10.1080/21655979.2021.1924554 (PMC8806528; doi:10.1080/21655979.2021.1924554)
Supplement: Supplemental Material [file KBIE_A_1924554_SM4728.zip › Document.rtf]

Supplementary  Figure legends
Figure S1. Pan-cancer analysis from TCGA dataset analyzed the expression levels of
(A) LINC00649 and (B) YAP1 mRNA in the STAD clinical tissues. (C) High-expressed
YAP1 mRNA predicted a worse prognosis in STAD patients.
