# Supplementary figures and images for: Long non-coding RNA LINC00649 regulates YES-associated protein 1 (YAP1)/Hippo pathway to accelerate gastric cancer (GC) progression via sequestering miR-16-5p
Source: Bioengineered. 2021 May 11;12(1):1791–802. doi: 10.1080/21655979.2021.1924554 (PMC8806528; doi:10.1080/21655979.2021.1924554)

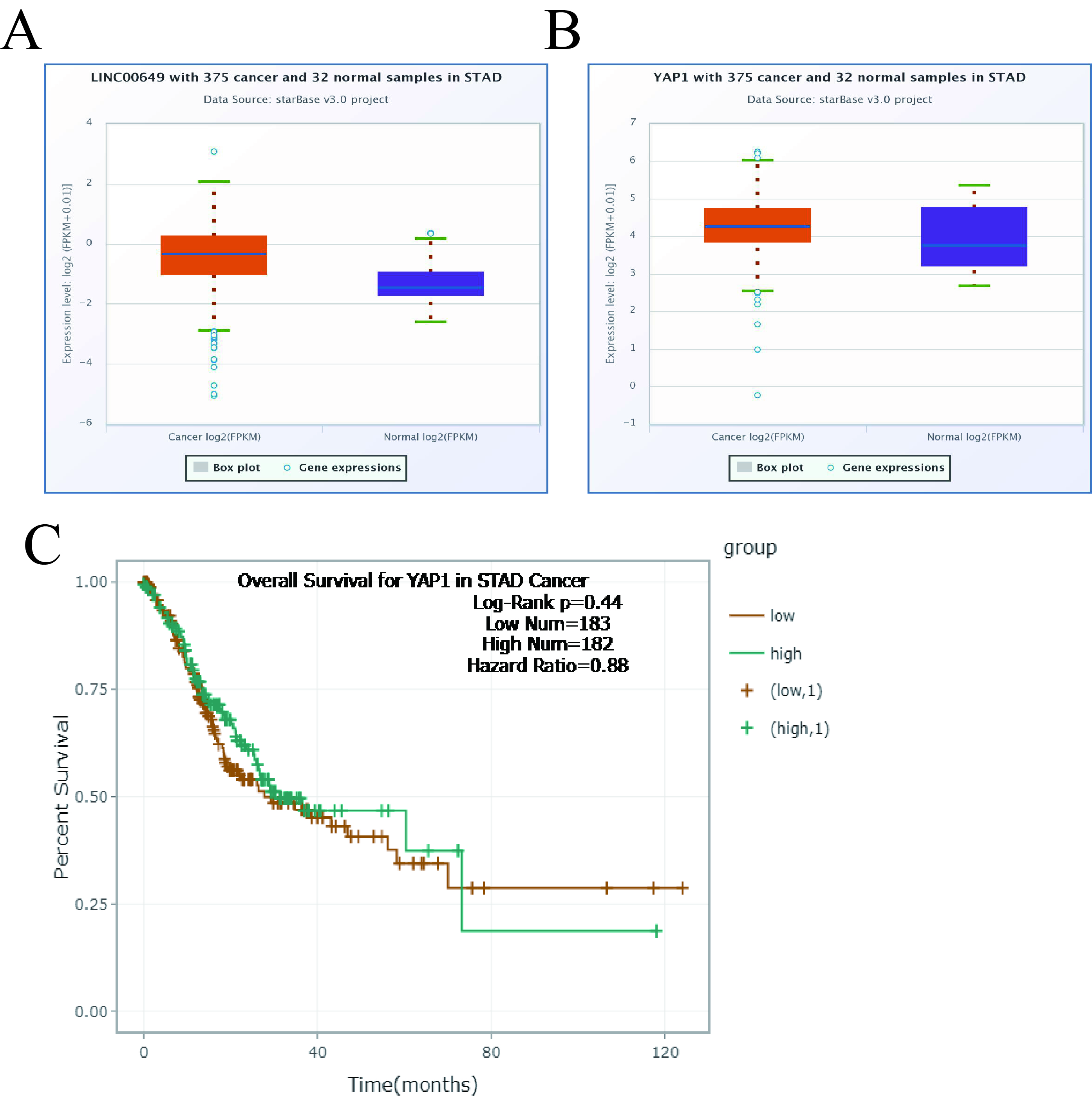

Supplement: Supplemental Material [file KBIE_A_1924554_SM4728.zip › Figure S1.jpg]

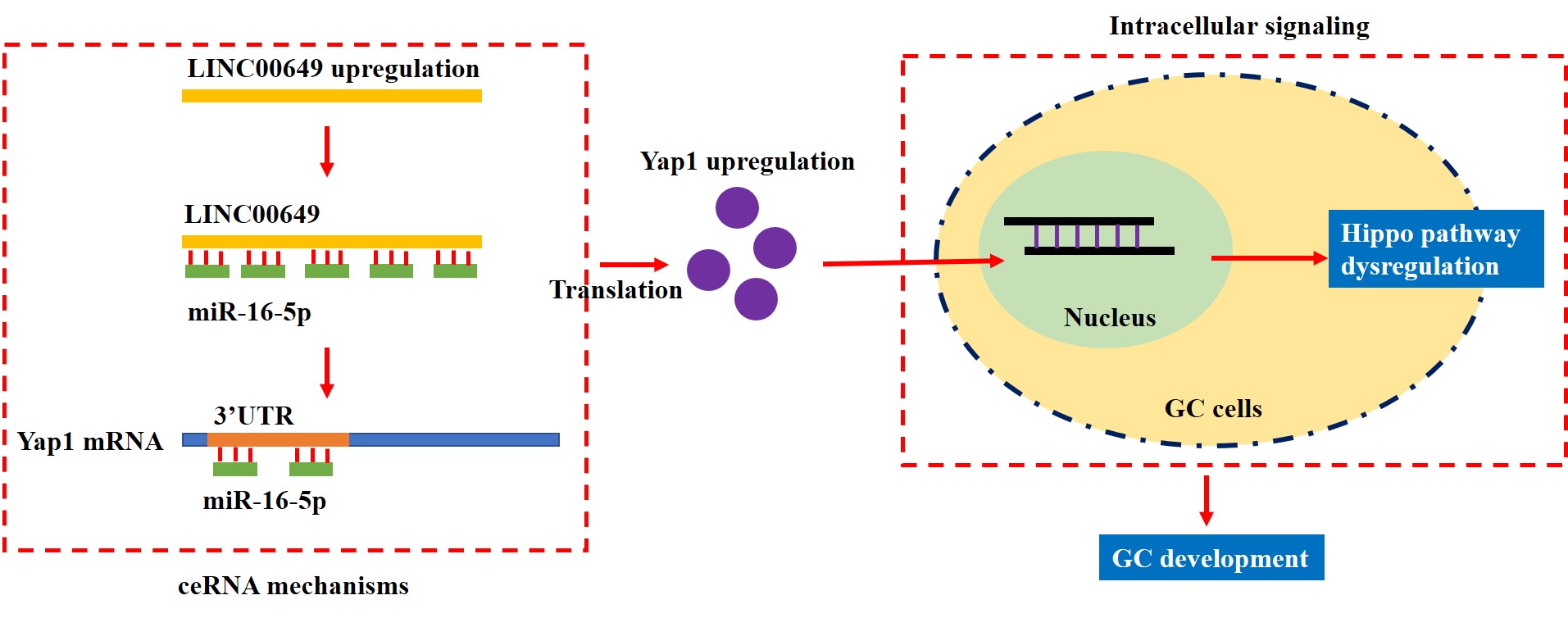

Supplement: Supplemental Material [file KBIE_A_1924554_SM4728.zip › Graphic Abstract.jpg]
